# Study Protocol - Assessing the feasibility of ‘Screening by Accredited Social Health Activist (ASHAs) and diagnosis by Community Health officers of Asthma and COPD at the primary health care System- A Novel Strategy- *SHVASAN*’

**DOI:** 10.3389/frhs.2026.1818244

**Published:** 2026-07-09

**Authors:** Ashwini Kishor Devane, Jayashree Sachin Gothankar, Prakash PrabhakaRrao Doke

**Affiliations:** 1 Central Research and Publication Unit (CRPU), Bharati Vidyapeeth (Deemed university) Medical College, Pune, India; 2 Department of Community Medicine, Bharati Vidyapeeth Deemed University Medical College, Pune, India

**Keywords:** ASHA (Accredited Social Health Activist), chronic respiratory diseases (CRDs), community health officers (CHOs), Feasibility Index, primary health care, *SHVASAN*, screening, diagnosis

## Abstract

*Underestimating the burden of Chronic respiratory diseases (COPD-Asthma) is a past error that needs to be remedied, and cases with these diseases are to be screened and diagnosed!* Current health policies primarily focus on other NCDs like diabetes and cardiovascular diseases, indicating a need for more targeted interventions for CRDs. A thorough literature review was conducted to analyse the evidence for probable solutions to address the CRD's and available models for screening and diagnosis at primary health care. Based on this, an implementation model is proposed- Screening by Accredited Social Health Activist (ASHAs)and diagnosis by Community Health officers (CHOs)of Asthma and COPD at the primary health care System- A Novel Strategy- ***SHVASAN*** (local meaning breathing).

**Objective:**

To assess the feasibility of implementation of a “***SHVASAN***” model for screening and diagnosis of COPD and Asthma at the primary health care level. Methodology: Mixed methods to assess feasibility, including the Delphi technique. Implementation will begin with the training of ASHAs, CHOs, and equipment will be provided for setting up the screening and diagnosis across one rural PHC in Pune district, India. All 35 villages and ASHAs would be screening the population above 30 years of age. And CHOs across all Health and wellness centres (06 + 02 at PHC and one at RH) will diagnose the CRDs. Feasibility would be assessed after one year of the ***SHVASAN*** model implementation. A Delphi study would be conducted to formulate the feasibility dimensions and formulate the feasibility index. Considering feasibility dimensions, qualitative and quantitative data would be collected to assess the feasibility of the model, which includes the population screened by the ASHAs and the number of diagnostic tests conducted at health wellness centers. And focused group discussions, in-depth interviews will be conducted to understand the facilitators and operational challenges of the ***SHVASAN*** implementation.

**Discussion:**

***SHVASAN*** builds on the NPCDCS Govt of India guidelines. This is a novel, low-cost adaptation for peripheral workers with limited specialist access.​This research will inform potential feasibility and the development of implementation strategies as public health services prepare for broader rollout in the near future.

**Clinical Trial Registration:**

CTRI/2025/10/095547.

## Introduction

Chronic respiratory diseases (CRDs), especially chronic obstructive pulmonary disease (COPD) and Asthma, are rapidly emerging public health problems in India. Recent pooled estimates suggest that the prevalence of COPD in India to be 7.4%, and community studies have reported a prevalence of COPD as 8.6% asthma approximately 3.30% to 8.7% (1–3). Although India contributes only 13% to the global asthma burden, it accounts for a disproportionately higher share of asthma deaths of 43%, indicating persistent gaps in timely diagnosis, access to appropriate treatment, and health system responsiveness (4, 5). Maharashtra state also contributes considerably to India's burden of CRDs in terms of prevalence (10% of India's CRD burden), deaths, and disability adjusted life years (DALYs), suggesting the need for specific strategies for early detection and correct management (6).

CRD burden extends beyond clinical morbidity. The development of COPD is slow and insidious, and symptoms tend to be noted by patients only after there has been a significant loss of lung function, often to 50-60% of the predicted value of peak expiratory value. It imposes substantial costs on patients, families, communities, and the health care system (7). The cost includes the cost for screening, diagnosis, pharmacological treatment, and management of exacerbations, hospital admissions, travel, and loss of productivity. The COPD cases reach the caregiver in the late stage of the disease when the dyspnoea is noticeable or when hospitalization is required. Delayed diagnosis further amplifies the economic burden, leading to considerable out-of-pocket health-care costs for severe COPD (7). People with COPD often present far too late to their doctor because they accept cough or mild breathlessness as a ‘normal’ result of aging, years of smoking, or exposure to biomass fuel (8).

The economic implications of delayed diagnosis and inadequate management are considerable. *Out-of-pocket expenditure for COPD in India is very high and is largely driven by hospitalization costs and drug costs* (9).The National Commission on Macroeconomics and Health (2005) estimated a substantial economic burden attributable to COPD and Asthma in India, emphasizing the need for public health approaches that move beyond hospital-based care. In resource-limited settings, this burden is likely compounded by limited access to diagnostic services, poor continuity of care, and delayed referral. Community-based screening by community health workers and early diagnosis may therefore offer an important opportunity to reduce avoidable morbidity, prevent exacerbations, improve quality of life, and may reduce long-term economic losses.

Considering the rising burden of chronic respiratory diseases, prevention and management guidelines for COPD and asthma were included in 2021 under the National Program for Prevention and Control of CVD, diabetes, cancers, and Stroke (NPCDCS) (10). It primarily focuses on non-communicable diseases (NCDs), such as diabetes and cardiovascular diseases, while underscoring the need for more targeted interventions for CRDs (10–12, 14). The NPCDCS includes screening for COPD at district hospitals but lacks comprehensive care at peripheral health centers near to patients’ home. The guideline specifically mentions the roles of primary health centers (PHCs) and Health and Wellness Centers (HWCs), ASHAs, and auxiliary nurse-midwives (ANMs) (13, 14). In many public sector settings, spirometry is available mainly at district hospitals, making confirmatory diagnosis inaccessible to suspected cases, who are often elderly persons from rural areas. This creates a critical diagnostic gap between community-level symptom recognition and facility-level confirmation.

The primary health care level, especially the HWC, offers an opportunity to bridge this gap by bringing the CRD screening and diagnosis closer to the community. With appropriate training and supportive supervision, the ASHA workers can identify the suspected CRD patient by measuring their peak expiratory flow rate. The Peak Expiratory Flow Rate (PEFR) has demonstrated effectiveness in suspecting conditions such as COPD and asthma, particularly in resource-limited settings (12, 15, 16). It can be proposed that a spirometry technician or ANM be trained to conduct spirometry tests at health and wellness centers (HWC), and that the community health officer (CHO) in charge of the HWC may interpret the results and diagnose CRDs after intensive training and telemedicine support for interpretation of the report. Provision of diagnostic (spirometers) and drugs at the health and wellness center will be desirable, as mentioned in the district program implementation plan by the state government (12, 17, 18)

Evidence from international and primary care models supports the feasibility of this approach. Brazil's Family Health Strategy (FHS) and South Africa's PACK program have demonstrated the value of integrated, guideline-based, team-delivered care for the chronic disease, including asthma and COPD (19, 20). The Global Alliance against Chronic Respiratory Diseases (GARD) has also emphasized strengthening health systems to prevent and control CRDs through primary health care (21). In India, two models are implemented for the screening and diagnosis of the CRDs through the primary health care. The SWAAS (Stepwise approach to airway diseases) program has demonstrated that active screening and early management of airway disease at the primary health care level can be feasible and potentially a cost-saving alternative to the current passive approach (22). Similarly, community-based initiatives using portable point-of-care spirometry in the Nashik district of Maharashtra's Dindori block have demonstrated operationally feasible, large-scale screening, diagnosis, and management of COPD and other chronic respiratory conditions outside tertiary care settings (23).

Together, these findings support the suggestion that a decentralized, primary care-based model for CRD screening and diagnosis may help address delayed diagnosis and improve access to chronic care in rural settings and emerge as a logical strategy for facilitating early detection of CRDs. However, evidence on the feasibility, implementation logistics, and operational evaluation of such models in routine primary health care settings remains limited.

The existing scientific literature provides substantial evidence to support the hypothesis that following a structured implementation model may be successful, which includes integrating community-based screening by Accredited Social Health Activists (ASHAs) using peak flow meters (12, 15, 16). and diagnostic confirmation at Health and Wellness Centres (HWCs) via spirometry. This model will enhance early detection and contribute to the strengthening of primary health care guidelines for COPD and asthma management. Although current CRD guidelines at the primary care level offer a supportive framework for such an intervention, the proposed model is poised to provide greater accessibility compared to existing approaches. The comparison of the guidelines and the proposed model is described in Table 1. With guidelines in place, yet a lack of clear implementation strategies at the ground level, it offers a timely and strategic opportunity to rigorously explore the feasibility of this model.

**Table 1:** The comparison of existing guidelines and the proposed ‘***SHVASAN***’ model.

Health facility	Key person involved	Existing guidelines Activities National Program for Prevention and Control of Cancer, diabetes, cardiovascular diseases, and Stroke, 2021 (14)	Proposed ‘***SHVASAN***’ model Activities
Beneficiary -Age 30 years and above			
At the community level	ASHA	Fill up a community-based assessment (CBAC)checklist for the above 30-year-old population with two relevant questions for risk factors of COPD Referral for the at-risk population to HWC- PHC and for other NCDs	- ASHA fill up the Risk Assessment for COPD and Asthma form for the above 30-year-old population - Mmrc - Cough assessment - PEFR measurement - Referral of suspected at HWC - Follow up of the patient put on treatment for inhalation techniques when diagnosed
Health and Wellness Centre	Community Health Officer (CHOs)	Questionnaire (assessment of cough and dyspnea) mMRC Health promotion for BCC Awareness generation and prevention of risk factors with a special focus on the cessation of smoking and exposure to pollutants/allergens Follow up on patients put on treatment and ensure treatment compliance and lifestyle modifications	- Cough assessment - mMRC - CAT score, R/S examination - Spirometry testing for diagnosis - Grading of disease severity and referral to PHC - Presence of co-morbidities - History of previous exacerbation - Coordination and support for prescription via telemedicine - Counselling for non-pharmacological interventions - Counselling for inhalation techniques when diagnosed - Referral in case of severe cases
Primary healthcare level	Medical Officer	- PEFR testing - If PEF < 80% predicted + breathlessness/cough>8 weeks or history compatible with Asthma, provisional diagnosis of Asthma - Individuals with PEF>80% of predicted, with cough and or dyspnoea should be assessed for TB and managed accordingly, and be educated on cessation and not starting smoking, weight reduction, nutrition, and physical activity. Individuals with PEF>80% of predicted, without cough and or dyspnea, may be sent back to the community after providing health education and risk reduction	- Telemedicine for diagnosed cases at HWC with the help of experts - Cough assessment - mMRC - CAT score, R/S examination - Spirometry testing for diagnosis - Grading of disease severity and referral to PHC - Presence of co-morbidities - History of previous exacerbation - Coordination and support for prescription via telemedicine - Counselling for non-pharmacological interventions - Counselling for inhalation techniques when diagnosed - Referral in case of severe cases
Equipment and tools are available at various levels			
At the Community Level		Community-level assessment tool by ASHA for 30 < population No equipment	Peak flow meters with mouthpieces A comprehensive questionnaire to detect the suspected cases Pulse oximeter Stadiometers Weighing machine Placebo kit to demonstrate the inhalation technique to the diagnosed cases
At Health and wellness		No equipment A tool for cough and dyspnoea mMRC	Spirometers Peak Flow rate meters Pulse oximeter Screening tool Placebo kit to demonstrate the inhalation technique to the diagnosed cases
At the Primary Health Centre		Peak flow meter	Spirometers Peak Flow rate meters Pulse oximeter Screening tool Placebo kit to demonstrate the inhalation technique to the diagnosed cases

### The *SHVASAN* model

The proposed structured ***SHVASAN*** model is thus conceptualized as a decentralized, primary health care-based approach for the screening and spirometry confirmation of the rural adult residents with suspected chronic respiratory diseases, namely COPD and asthma. The model is based on evidence from national guidelines that recognize the role of the primary health care platform and ASHA in the prevention and control of NCDs; the second evidence is based on the international primary care model; and the third evidence is from the Indian models of Kerala and Maharashtra. The proposed model is named ’Screening by Accredited Social Health Activist (ASHAs) and diagnosis by Community Health officers of Asthma and COPD at the primary health care System (***SHVASAN***), is therefore designed to assess the feasibility of integrating decentralized community-based screening using a pretested validated questionnaire, and peak expiratory flow assessment by trained ASHA workers, referral support within the primary health care system, and spirometry evaluation by CHO at HWC, with the help of a trained technician. By evaluating this model in a rural setting, the study aims to generate evidence that may inform scalable strategies for early detection and management of COPD and asthma.

**Figure 1:**
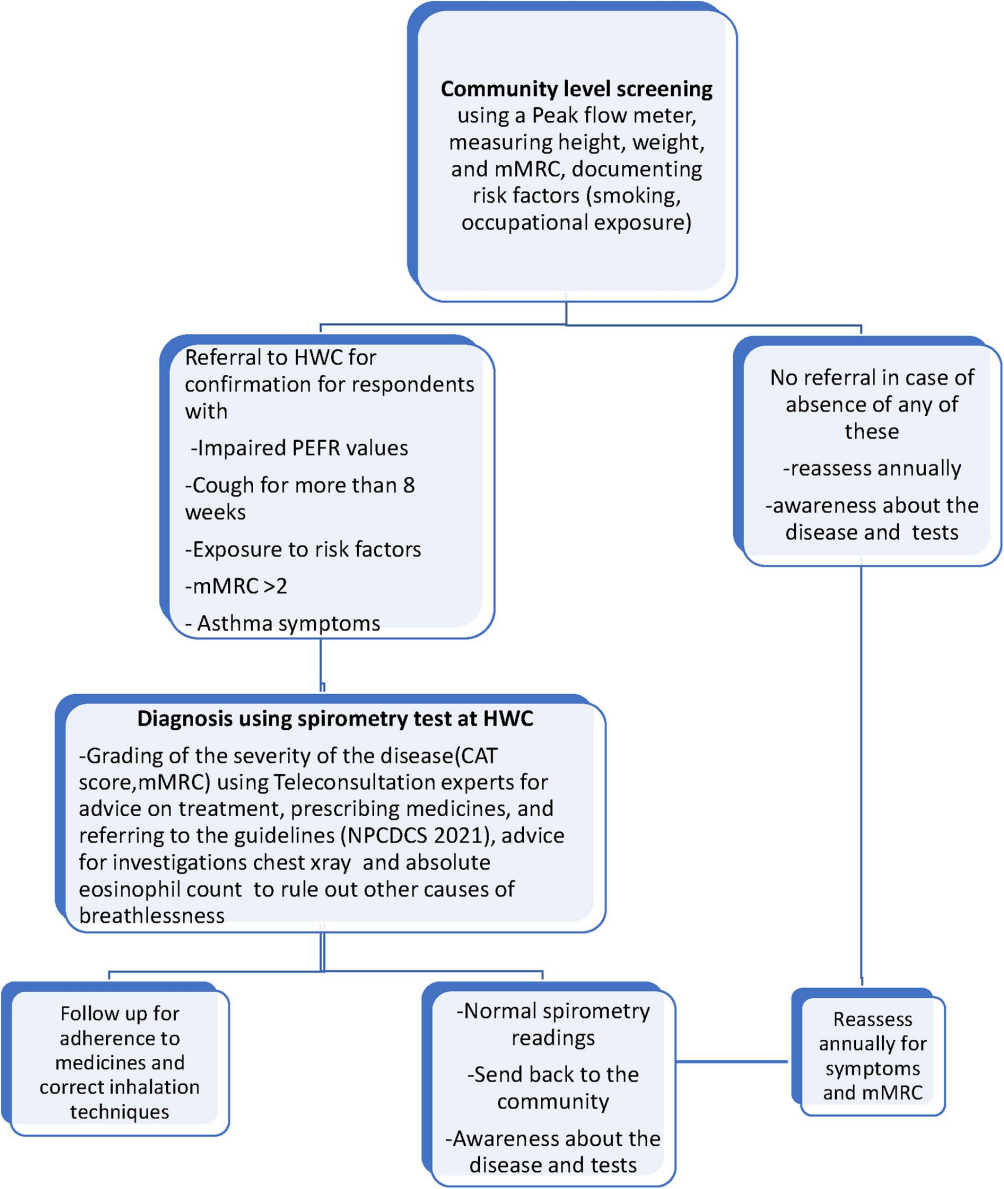
The ‘***SHVASAN***’ model.

The primary objective of the proposed study is to assess the feasibility of implementing the ‘***SHVASAN**’* model as a primary health care-based approach for screening and diagnosis of COPD and Asthma. The details of the ***SHVASAN*** model are given in Figure 1. The secondary objectives are to identify implementation facilitators and operational challenges, and to compare the proportions of newly diagnosed COPD and Asthma cases among adults aged 30 years and above between the intervention PHC and the adjacent non-intervention PHC.

The comparison delineates the scope of the proposed model, suggesting that, based on the government guidelines, with capacity building of manpower and upgradation of existing infrastructure, the screening and diagnosis may be feasible. (Table 1)

## Methods and analysis

### Study design

This will be a mixed-methods implementation study to assess the feasibility of the ***SHVASAN*** model for screening and diagnosing COPD and asthma at the primary health care level.

The study will include the following components:

First, a longitudinal quantitative component in the implementing PHC to assess the feasibility of implementing the ***SHVASAN*** model over the study period from the initial implementation. Implementation will begin with the training of ASHAs, CHOs, and equipment will be provided for setting up the screening and diagnosis across the Male PHC in Pune district, India. All 35 villages and ASHAs would be screening the population above 30 years of age. And CHOs across all Health and wellness centres (06 + 02 at PHC and one at RH) will diagnose the CRDs. The longitudinal quantitative component will include the number screened, number suspected, number referred, number reached the HWC, number underwent and received a diagnosis, number put on treatment, and number followed up. Second a Delphi technique to obtain expert consensus on the following components: Technical feasibility, Operational feasibility, resource feasibility (including indicators), Measurement strategy, measurement scoring, and appropriate data sources for each feasibility dimension, and finalising ***SHVASAN*** characteristics. This involves finalizing the intervention package and assessing the expert opinion for feasibility indicators. The third qualitative component is embedded in the longitudinal quantitative component. This includes a focused group discussions (FGDs), which will be undertaken among the key stakeholders to inform quantitative implementation of the ***SHVASAN*** model. Fourth, the analytical cross-sectional component will be conducted to compare the proportions of newly diagnosed COPD and asthma cases among adults aged 30 years and above over one year between the implementing and the adjacent PHC using routine Health Management Information System (HMIS)data. This component will help assess whether implementing the ***SHVASAN*** model is associated with improved case detection for COPD and asthma compared to routine NCD implementation. The explanatory sequential component with qualitative methods to explore the facilitators, barriers, acceptability, accessibility, and operational challenges associated with the implementation of the ***SHVASAN*** model, including the stakeholders such as medical officers (MO), community health officers (CHO), ASHA, and participants above the age of 30 years.

### Study setting

**Figure 2:**
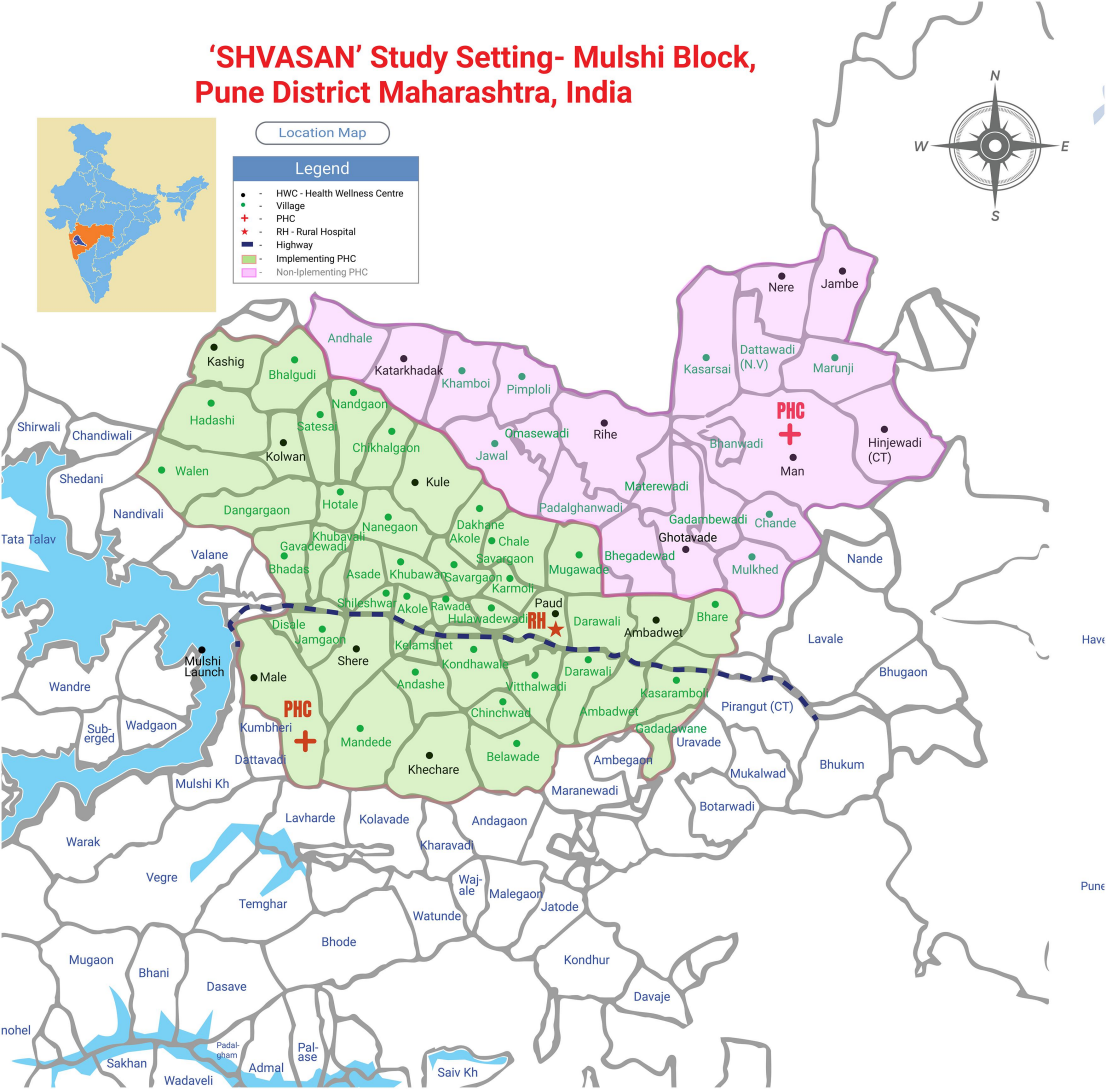
‘***SHVASAN***’ study setting- Mulshi Block, Pune District Maharashtra, India.

The study will be implemented in the Mulshi block (Figure 2), Pune district, Maharashtra. The Pune district is situated in western Maharashtra, at the foothills of the Sahyadri ranges, and exhibits marked geographical heterogeneity. It is one of the high-rainfall talukas of Pune district and is part of the western hilly region. Paud village in the block is the administrative headquarters. The public health infrastructure in Mulshi includes one rural hospital in Paud and four primary health centers in *Male,* Maan, Mutha, and Ambavane (24).

The implementing site will be the *Male* PHC*,* where the ***SHVASAN*** model will be implemented and assessed for feasibility. The total population of *Male* PHC is 12,148 adults aged 30 years and above residing in 35 villages. The health care infrastructure includes eight HWCs, including one at the primary health center and one at the Paud rural hospital. An adjacent PHC from the same block, namely *Maan* PHC, will be selected as a non-implementing PHC for the secondary comparative objective. The Maan PHC is similar to Male PHC with respect to geographic, socio-demographic, cultural, environmental, and health care system context. It will continue routine providing services and will be used only to compare the proportions of newly diagnosed COPD and asthma cases among adults over a one-year study period.

### Study duration

**Figure 3:**
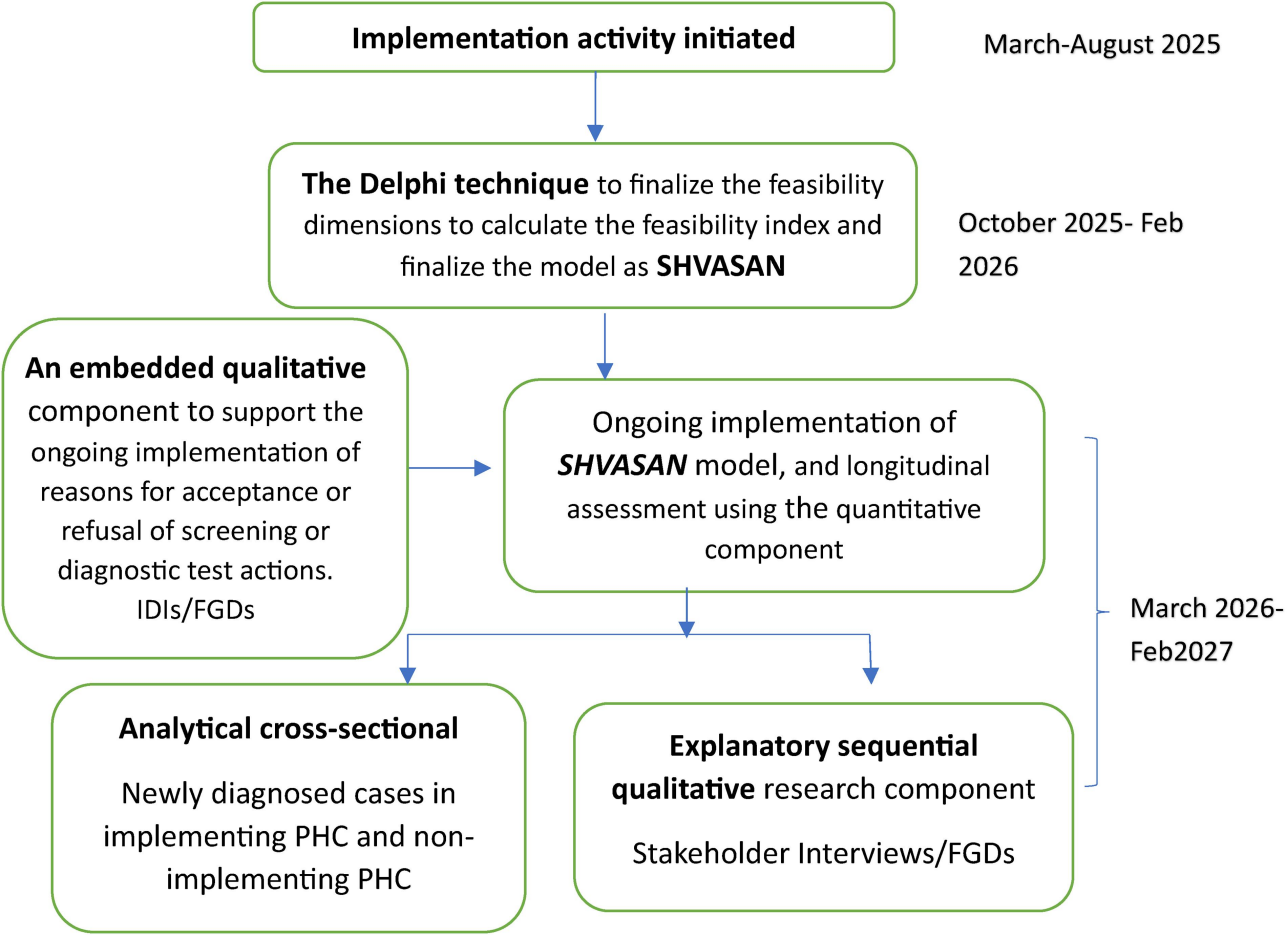
Sequence and timeline of various designs used in the proposed study.

The total study duration will be two years, from March 2025 to Feb 2027, following approval from the ethics committee and permission from the block and district-level authorities. The timelines and study design are mentioned in Figure 3.

### Methodology to measure feasibility index

A feasibility assessment will help determine whether the research is practical, manageable, and likely to achieve the screening and diagnosis of COPD or asthma. Little is known about the feasibility of screening and diagnosing COPD-Asthma in peripheral health care delivery; therefore, to determine the feasibility measure, a unique methodology will be applied. The Delphi Method: Delphi's value for adaptation, promoting feasibility metrics and iterative refinement to connect the evidence-to-practice gaps in primary care (25). The Delphi method is most suitable as it is a structured, iterative process used to gather and refine the opinions of a panel of experts to finalize the feasibility measures, focusing on assessing feasibility (26). Based on the Delphi method output, a feasibility index may be developed at the health and wellness center level. The final dimensions later guide the questionnaire for the qualitative and quantitative data collection.

The Delphi technique seeks to address the following question related to the Feasibility Index of the ***SHVASAN*** model:

(a) Are the proposed dimensions for assessing feasibility appropriate?
(b) What suggestions would you have to improve the dimensions? (By adding the new dimension, by deleting the suggested dimensions, or by modifying the dimension)
(c) How can the feasibility index be developed based on the given dimensions?
(d) How can each component be weighted? (Table 2)

The steps for the Delphi technique:

(a) Draft feasibility dimension and implementation indicators for the ***SHVASAN*** model based on literature review, program guidelines, and field requirements
(b) The experts were purposively selected and invited based on their domains and expertise in the field of implementation research, health system and policy researchers, policy implementers, health administrators, public health academics, and pulmonology. A panel of six to eight experts will be targeted to ensure multidisciplinary representation.
(c) Sharing of the first round of the Delphi questionnaire/related material with the shortlisted feasibility dimensions, asking them their opinion based on relevance, feasibility dimensions suitability, they will be asked to modify (MO) or keep (KP) the dimension, and share feedback on weights to each feasibility component.
(d) Collect and summarize the first feedback using descriptive statistics and synthesis of open-ended responses, if any.
(e) Revise, merge, delete, or add items based on the first round
(f) A consensus of >95% agreement among the participating experts for retaining the dimension will be considered.
(g) An iterative round to share feedback and revised items in the second round of the Delphi technique.
(h) Analysis of second round expert feedback to identify consensus and non-consensus items.
(i) Finalize the feasibility dimensions and weights to calculate the Index.
(j) Final dissemination meeting with Experts

**Table 2:** The proposed feasibility dimension to calculate the index includes.

Feasibility Component	Technical feasibility	Operational feasibility	Resources Feasibility	
Indicators	1. Recruitment rate 2. Quality of Data Collection Procedures 3. Outcome Measures 4. Technical skill in performing the PEFR 5. Suspected cases- referrals from the screened population 6. Technical skill of conducting a spirometry test 7. Correct interpretation and diagnosis	1. Overall stakeholder acceptability (Participant satisfaction Staff satisfaction, Readiness to perform screening and diagnosis techniques) 2. Month-wise percentage of time taken for the ASHAs to complete their target 3. Month-wise percentage of time taken by CHOs for the screening and diagnosis 4. percentage of deviation in the place of conduction (actual vs. deviation) 5. Dropout Rates/Retention Rate	1. Infrastructure to deliver the Model 2. Human resources 3. Cost for equipment Direct Indirect 4. Cost for training 5. Cost per participant	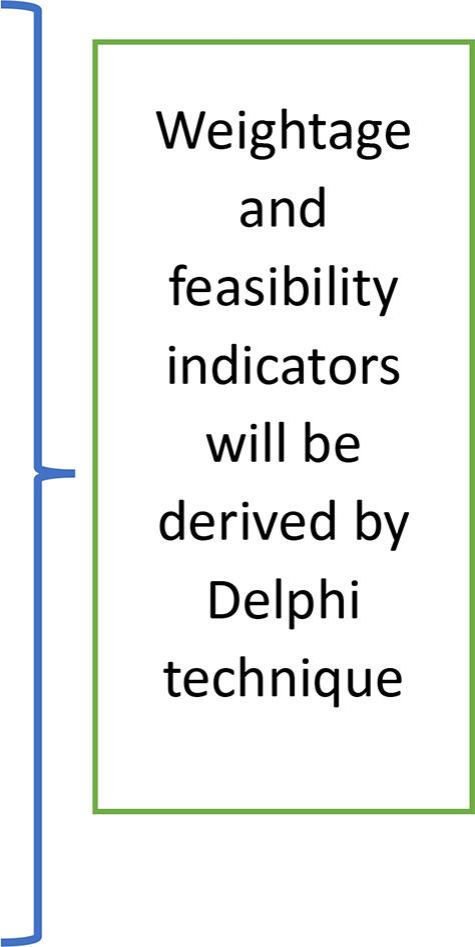

### Quantitative component

Sample Size estimation for quantitative component: The sample size calculation is based on an estimated prevalence of COPD of 8% (1, 2). Considering an allowable error (d) of 0.8% (10% of the prevalence) and a 95% confidence level, the required sample size for the study is 4,418 adults. For the non-probability sampling technique, a design effect of 2 is applied to account for potential loss to follow-up. Thus, the final sample size is 8,836 adult participants.

Inclusion criteria – Permanent residents, are of age 30 years and above, and are ready to consent. Implementation stakeholders (ASHA, CHOS, MO, Community representatives from implementing PHCs, Taluka health officer, District health officer) are ready to consent

Exclusion criteria: Terminally ill adults

Research Tools: Quantitative tools will be used to calculate the feasibility index for the ***SHVASAN*** model.

1. A Feasibility index document tool is being developed using the Delphi technique. That will include feasibility dimensions: Technical, Operational, and Resource- All three dimensions would be measured using study tools. The COPD-Asthma screening questionnaire: Existing pretested and validated questionnaires assessing the risk factors and exposures, such as smoking, use of biomass fuel, family history, quality of life, and medical history, as well as known cases of COPD or Asthma in the eligible population. The Peak exploratory flow rate (PEFR) skills, screening of the population, will help calculate the technical feasibility. That may be reflected through the number of suspected cases, the recruitment rate, the retention rate, and the screening rate.
2. The COPD-Asthma diagnosis proforma: An existing, pretested, and validated proforma will be used to diagnose suspected cases. It will be filled by CHO at HWC. The proforma will document the medical history, symptoms, comorbidities, treatment, and vital clinical measurements such as blood pressure, blood oxygen level, Pulse, etc. Related and other pertinent clinical findings will be noted. A pre- and post-spirometry reading and interpretation in accordance with the NPCDCS 2021 guidelines, the categorization of COPD and asthma will be documented in the same proforma. This will also help calculate the technical feasibility of the diagnosis.
3. The PEFR checklist for skills assessment of ASHAs would be completed by the investigator at two time points: after the training and during field data collection by ASHAs.
4. Spirometry testing activity checklist for the skill assessment during the conduction of the test at HWC.

Data Quality Checks

1. Data collection –for the quantitative data, Quality checks will be conducted while collecting data at the community level. Quality checks would be conducted randomly on at least 5% of the sample.
2. Quality checking for the Peak flow meter skill can be done using the checklist to be filled out by the field senior supervisors
3. A Spirometry quality checklist will also be filled out every time of the observation.
4. Random quality checking visits will be planned by technical experts for PEFR and Spirometry testing.

### Qualitative component

Sample size for the qualitative component: Purposive sampling will be used to include stakeholders involved in implementing the ***SHVASAN*** model and its beneficiaries. The participants are mentioned below (Table 3). The relevant stakeholders from the adjacent PHC will be involved to understand the routine practices and the contextual differences. The data collection will continue until the saturation is reached.

**Table 3:** Stakeholders for ***SHVASAN*** model-.

Stakeholders	Role in ***SHVASAN*** implementation	Data collection methodology
Accredited social health activists (ASHAs) from implementing and non-implementing PHCs ASHA supervisors	Screening of participants aged 30 years and above, who routinely do NCD screening activities at the village level Monitoring of data collection and field visits for quality assurance	Focused group discussion In-depth interviews
Participants aged 30 years and above	Beneficiaries of timely screening and diagnosis of COPD/Asthma	Focused group discussion In-depth interviews
Community health officers	Diagnosis of participants aged 30 years and above, who routinely do NCD management and diagnosis at health and wellness centres	In-depth interviews
Medical officers implementing PHCs and in non-implementing PHCs	Diagnosis and management of cases visiting the primary health centre/ referrals from Health and wellness centres	In-depth interviews
District Health Officer (DHO) and Block Taluka Health Officer (THO).	Administrators from health systems who share permissions facilitate smoother functioning	In-depth interviews
Local village leaders	Local facilitators for smoother implementation	In-depth interviews

Qualitative tools: the following qualitative tools will be created, validated, and used.

(a) Focus Group discussion guide for ASHAs and an in-depth interview guide for ASHA Supervisor
(b) Focus Group discussion guide Participants (PEFR testing, spirometry diagnosed, spirometry non-diagnosed)
(c) In-depth interview guide for CHO
(d) In-depth interview guide for Medical Officer
(e) In-depth interview of the local village leaders
(f) In-depth interview guide for administrators, namely District Health Officer (DHO) and Block/Taluka Health Officer (THO).

Quality checks for the qualitative component

1. Ensures data triangulation with data collection from multiple levels of stakeholders.
2. Feeding back data, analytical categories, interpretations, and conclusions to stakeholders such as participants, ASHAs, CHOs, etc.
3. The records of the research path are kept throughout the study, from documentation for prolonged engagement with the community and stakeholders to audio files, transcripts, and final reporting.

The analytical cross-sectional component

This is to compare the proportions of newly diagnosed COPD and asthma cases among adults aged 30 years and above over one year between the implementing and the adjacent PHC using routine Health Management Information System (HMIS)data. This component will help assess whether implementing the ***SHVASAN*** model is associated with improved case detection for COPD and asthma compared to routine NCD implementation.

The data for diagnosis in implementing and non-implementing PHCs would be accessed from the Public Health Management Information System (HMIS).

Plan for analysis

1. Quantitative - Data will be entered in Microsoft Excel and then imported to SPSS (version 27) for Windows package (IBM SPSS Statistics for Windows, Version 25.0. Armonk, NY: IBM Corp). The proportions of categorical variables of interest (Respondents with COPD-Asthma diagnosis in both PHCs) will be compared using the Chi-Square test. Means of variables of interest (time for symptoms, diagnosis, age, severity of disease) will be compared using the independent t-test. The associations between disease proportion and PEFR and spirometry values will be determined using the chi-square test. A multivariate logistic regression analysis will be carried out to identify significant predictors using PEFR values in implementing PHCs.
2. Qualitative analysis -Data from IDIs and focus group discussions will be transcribed and thematically analyzed, and if required, MaxQDA software may also be used. In-depth interviews and focus group interviews will be transcribed verbatim, checked, and corrected if required. The transcripts will then be thematically analyzed. The transcripts will be independently read to become familiar with the material. The notes on insights and patterns from the transcripts will be derived. Subsequently, participants and/or the guide may engage in discussion to reach a consensus on the content. Coding transcripts using code labels closely linked to the meaning of the quotes, and organizing codes with similar content into groups and themes, would generate initial themes. Groups and initial themes were then presented and discussed with the guide. Then, if suitable, the Normalisation Process Theory (NPT) (27) will be used as a theoretical lens to understand how the implementation of codes and initial themes, organized within the constructs of NPT, were reviewed before being refined and delineated into four main themes. In cases where themes overlap with more than one NPT construct, a best-fit choice will be made. The content will be finalized thematically later.
3. The comparative analysis of data from both PHCs would be done using the chi-square test for characteristics of diagnosed cases. The number of newly diagnosed cases during the study period would be obtained from the Public Health Management Information System (HMIS)
4. Feasibility index - Based on qualitative and quantitative data collection and analysis, the feasibility dimensions will be scored and finalized through Delphi consensus. The feasibility index may be derived from the scores and the assigned weights.

### Outcomes

#### Primary outcome

Feasibility of implementing the ***SHVASAN*** model by calculating the feasibility index for the feasibility of ’Screening by Accredited Social Health Activist (ASHAs)and diagnosis by Community Health officers of Asthma and COPD at the primary health care System- A Novel Strategy (***SHVASAN*** model). This will consider technical, operational, and resource feasibility.

#### Secondary outcome

Secondary outcome: Facilitators and operational challenges in the Implementation of the ‘***SHVASAN***’ Model. Prevalence of COPD and Asthma in implementing PHC and in adjuvant adjacent PHC.

## Discussion

***SHVASAN*** builds on the COPD 2021 guidelines by utilizing peak flow meters for scalable ASHA-led screening. This is a novel, low-cost adaptation for peripheral workers where specialist access is limited.​Unlike hospital/clinical-centric models, it integrates history-taking, risk assessment, and portable simple peak flow meter (15) portable spirometry at HWCs, close to the successful Kerala SWASS model, but tailored for CHO training for diagnosis at HWCs.​ (22)Feasibility metrics (yield, cost, acceptability) are uniquely strong in their use of a mixed-methods approach. Successful ***SHVASAN*** implementation can inform the NCD 2023 expansion and strengthen policy recommendations as public health services prepare for broader rollout in the near future.

Limitations and Challenges: The generalizability of ***SHVASAN*** findings beyond Pune's rural PHCs hinges on multi-district scaling trials, as local dynamics may not fully translate across diverse Maharashtra contexts, such as tribal belts and coastal clusters. ASHA workload pressures in NHM settings pose a significant challenge, as community health workers often juggle multiple responsibilities alongside respiratory screening demands. Severe cases need referrals to a higher centre.

### Ethics and dissemination

#### Ethical considerations

The final study protocol, including the final version of the other essential documents, is approved by the Institutional Ethics Committee (DHR Reg. No: EC/New/INST/2022/MH/0150 – Ref No. BVDUMC/IEC/317/25-26). There is no risk involved in conducting the study. Written informed consent will be taken before voluntary participation in the study. The PEFR and spirometry procedures will be explained at the time of the tests and performed as per NPCDCS 2021 guidelines. Anonymity and confidentiality will be maintained throughout the study, using study identifiers by assigning participant codes.

### Dissemination

Sharing with policy shapers and makers

- Preparing action-oriented policy briefs that may highlight key findings, implications, and specific recommendations tailored to national, state, or district-level decision-makers.
- Engage with policymakers early and throughout the study (e.g., formal meetings, consultative meetings) and present findings through trainings/ technical briefings

Feeding back results to the community

- Organise community dissemination sessions (village meetings, or stakeholder conclaves) using local language, to explain what the study found

Presenting at scientific conferences

- Submit abstracts and present findings at national and international conferences to share methods, preliminary or final results, and lessons learned with other researchers.

Publishing in peer-reviewed journals-

- Papers on study design, methods, major findings, or implementation experience, and submit them to relevant peer-reviewed journals in public health, respiratory medicine, or primary care
